# Cefepime extended infusion versus intermittent infusion: Clinical and cost evaluation

**DOI:** 10.1017/ash.2023.179

**Published:** 2023-07-10

**Authors:** Aalok V. Khole, Emily Dionne, Emily Zitek-Morrison, Maureen Campion

**Affiliations:** 1 Division of Infectious Diseases and International Health, Cheshire Medical Center/Dartmouth Health, Keene, New Hampshire; 2 Department of Pharmacy, UMass Memorial Medical Center, Worcester, Massachusetts; 3 Department of Population and Quantitative Health Sciences, University of Massachusetts Chan Medical School, Worcester, Massachusetts; 4 Department of Pharmacy, Tufts Medical Center, Boston, Massachusetts

## Abstract

**Background:**

Extended infusion cefepime (1 gram every 6 hours administered over 3 hours) achieves pharmacodynamic efficacy against bacteria with a MIC of ≤8 mg/L in Monte Carlo simulations. This regimen has not been evaluated in clinical practice.

**Objective:**

Compare clinical and economic outcomes for cefepime by intermittent infusion and by extended infusion in the acute-care setting.

**Design:**

Single-center, retrospective cohort study.

**Setting:**

Tertiary-care academic medical center.

**Patients:**

Hospitalized adults who received cefepime between August 2016 and July 2018 with a diagnosis of sepsis or pneumonia.

**Methods:**

Clinical and economic outcomes were compared for patients who received empiric cefepime via intermittent infusion (30-minute infusion of 2 g every 8 hours) or extended infusion (3-hour infusion of 1 g every 6 hours). Clinical outcomes analyses were carried out using appropriate statistical methods.

**Results:**

Overall, 111 patients received intermittent infusion and 93 patients received extended infusion. Approximately half of the included patients had a positive culture for a bacterial pathogen (intermittent infusion 45.9% vs extended infusion 47.3%). Median hospital length of stay (intermittent infusion 6 days vs extended infusion 6 days; *P* = .67) and 90-day readmission rates (intermittent infusion 61.3% vs extended infusion 67.7%; *P* = .34) did not differ between the groups. Mortality was infrequent in both groups (intermittent infusion 2.9% vs extended infusion 1.5%; *P* = .45). Cefepime cost per patient was lower with cefepime by extended infusion: average total daily cost $86.06 for intermittent infusion versus $43.39 for extended infusion.

**Conclusions:**

Cefepime via extended infusion (4 grams/day) did not differ in clinical outcomes compared to intermittent infusion (6 grams/day) but reduced drug expenditure. Prospective, multicenter, high-quality studies should be conducted to evaluate a mortality difference between these regimens.

Multidrug-resistant gram-negative organisms, such as *Pseudomonas aeruginosa*, can cause severe infections like pneumonia and bacteremia, which constitute a tremendous burden on the healthcare system. Mortality rates associated with these infections range from 18% to 60% and have accounted for US$767 million in annual attributable healthcare costs over the last decade.^1–4^ Beta-lactam antibiotics, such as cefepime, are preferred in treatment regimens against these infections due their broad-spectrum coverage and relatively low resistance rates.^5^

Like other β-lactam antibiotics, cefepime displays time-dependent bactericidal activity, and its efficacy is enhanced when free drug concentrations exceed the minimum inhibitory concentration (MIC) for at least 60%–70% of the dosing interval. A variety of cefepime dosing regimens have been tested to optimize time above the MIC, including increased frequency of administration and extended infusions given over 3–4 hours. Extended infusions help achieve free drug concentrations that exceed the MIC for longer periods of time (ƒT > MIC).^6^ Studies have shown that extended infusions of β-lactam antibiotics reduce mortality, length of stay and overall healthcare costs.^1,2,5,7^ The dosing method of cefepime 2 grams every 8 hours administered over 3–4 hours has a high probability of achieving pharmacodynamic goals for *P. aeruginosa* with a MIC equal to 8 mg/L.^8^.^9^ Monte Carlo simulations have shown that administering cefepime 1 gram every 6 hours infused over 3 hours achieves similar pharmacodynamic goals.^10,11^ Although a regimen that utilizes 4 grams/day appears equally efficacious in Monte Carlo simulations, no studies have evaluated its use in clinical practice, to the best of our knowledge.^10^

UMass Memorial Medical Center (UMMMC), a tertiary-care academic medical center in Worcester, Massachusetts, changed its cefepime dosing strategy for empiric treatment of gram-negative infections, including *Pseudomonas aeruginosa*, from 30-minute intermittent infusions (6 grams/day) to 3-hour extended infusions (4 grams/day) in August 2017. This change was approved by the Pharmacy and Therapeutics Committee and Critical Care Operations Committee for all admitted adult patients. Extended infusions became the standard of care as ordered by providers or adjusted per protocol by pharmacists. In this study, we compared patient outcomes including length of stay, mortality, readmission rates, and medication costs for patients who received intermittent infusions and those who received extended infusions.

## Methods

### Study design

This was a single-center retrospective cohort study conducted at UMMMC. Patients who received cefepime between August 2016 and July 2018 were identified for review from a drug-use report in the Crimson Database (The Advisory Board Company, Washington, DC). The Crimson Database is a third-party database that includes information about diagnosis-related groups (DRGs), length of stay, mortality, and readmission. Patients were divided into 2 groups for comparison: those who received intermittent-infusion cefepime (30-minute infusion) and those who received extended-infusion cefepime (3-hour infusion). Prior to August 2017, all patients received intermittent infusion. After August 2017, all patients received extended infusion, although the first dose could be administered over 30 minutes. The dosing frequency was adjusted for renal function using the Cockcroft-Gault equation to calculate creatinine clearance (Tables 1 and 2). No other significant changes were made to the clinical management of sepsis or pneumonia during the study period. Ethics approval was granted by the UMass Chan Medical School Institutional Review Board (study no. H00016115).

**Table 1.** Institutional Dosing Recommendations According to Renal Function for Intermittent (30-minute) Cefepime Infusions

Renal Function^a^	Severe Infection^b^	Moderate Infection^c^
>60 mL/min	2 grams q8h	2 grams q12h
30–60 mL/min	2 grams q12h	2 grams q24h
11–29 mL/min	2 grams q24h	1 gram q24h
<11 mL/min	1 gram q24 h	500 mg q24h
HD	1 gram q24h	1 gram × 1 dose then 500 mg q24h
CVVH (effluent rate 2–2.5 L/h)	2 grams q12h	1 gram q12h

Note. q8h, every 8 hours; q12h, every 12 hours; q 24h, every 24 hours; HD, hemodialysis; CVVH, continuous veno-venous hemofiltration.

a Determined by Cockroft-Gault equation.

b Sepsis, meningitis, pneumonia, neutropenic fever, and *P. aeruginosa* infections.

c Intra-abdominal, skin and soft tissue, and pyelonephritis infections.

**Table 2.** Institutional Dosing Recommendations According to Renal Function for Extended Infusion (3 hour) Cefepime Infusions

Renal Function^a^	Meningitis	Severe Infection^b^	Moderate Infection^c^
>50 mL/min	2 grams q8h	1 gram q6h	1 gram q8h
30–50 mL/min	2 grams q12h	1 gram q8h	1 gram q8h
<30 mL/min	2 grams q24h	1 gram q12h	1 gram q12–24h
HD	2 grams q24h	1 gram q24h	1 gram q24h
CVVH (effluent rate 2–2.5 L/h)	2 grams q12h	1 gram q12h	1 gram q12h

Note. q6h, every 6 hours; q8h, every 8 hours; q12h, every 12 hours; q 24h, every 24 hours; HD, hemodialysis; CVVH, continuous veno-venous hemofiltration.

a Determined by Cockroft-Gault equation.

b Sepsis, pneumonia, neutropenic fever, and *P. aeruginosa* infections.

c Intra-abdominal, skin and soft-tissue, and pyelonephritis infections.

### Patients

Patients were included if they were aged ≥18 years, had a Medicare Severity-Diagnosis-Related Group (MS-DRG) of sepsis or pneumonia, and received cefepime for 48 hours or longer. Patients were excluded if they met any of the following criteria: organism isolated in bacterial culture was cefepime resistant; received another β-lactam antibiotic with activity against *P. aeruginosa* within 48 hours of cefepime therapy; received both intermittent-infusion and extended-infusion cefepime; transitioned to comfort measures only status; or due to incarceration, pregnancy, or fever in the setting of neutropenia with unknown source. Patients in the extended-infusion group were not excluded if their first dose of cefepime was administered over 30 minutes. Cefepime resistance was defined as a positive culture with an MIC ≥32 mg/L for *P. aeruginosa* or ≥16 mg/L for *Enterobacteriaceae* in accordance with CLSI (Clinical and Laboratory Standards Institute) guidance.^12^

### Data collection

Demographic and outcome data were collected from electronic medical records and Crimson Database (The Advisory Board Company, Washington, DC). Demographic data obtained included age, sex, and primary diagnosis during hospitalization. A chart review was conducted to determine white blood cell (WBC) count and serum creatinine (SCr) upon cefepime initiation, additional antibiotics for gram-negative coverage, days of cefepime therapy, microbiological data, and the presence of an infectious disease consultation. Severity level was calculated utilizing 3M™ All Patient Refined-Diagnosis Related Group (APR-DRG), which includes age, sex, primary and secondary diagnoses, procedures, status at discharge, and days on mechanical ventilator.^13^ Microbiological data included results of all blood, respiratory, urine, wound and intra-abdominal cultures collected. Antibiotic susceptibility testing was performed using Vitek2™ Systems (Biomerieux). Clinical outcomes data included: in-hospital mortality, hospital length of stay, ICU length of stay, and 30-day and 90-day hospital readmission. Cost of cefepime therapy was calculated based upon the hospital’s wholesale purchase price, patient-specific dosing, and duration of therapy.

### Statistical analysis

Statistical analyses were performed using SAS version 9.4 software (SAS Institute, Cary, NC). Categorical data were analyzed using the χ^2^ test or the Fisher exact test. Nonparametric continuous data were compared using the Wilcoxon rank-sum test. A 2-tailed *P* value ≤ .05 was considered statistically significant.

## Results

**Fig. 1.**
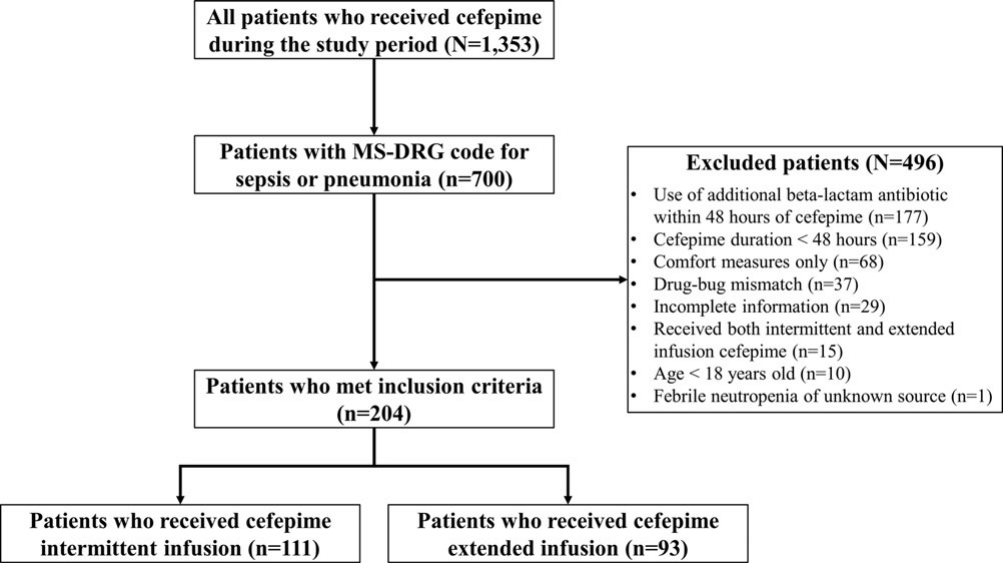
Flow chart of patients evaluated for study inclusion.

In total, 1,353 patients received cefepime during the study period. Of these patients, 700 had an MS-DRG code for sepsis or pneumonia upon discharge. Among the patients who had an MS-DRG code for sepsis or pneumonia, 496 patients were excluded (Fig. 1). The most frequent reasons for exclusion were concurrent use of another antibiotic with anti-*Pseudomonas* coverage within 48 hours of cefepime administration (n = 177) and cefepime duration <48 hours (n = 159).

**Fig. 2.**
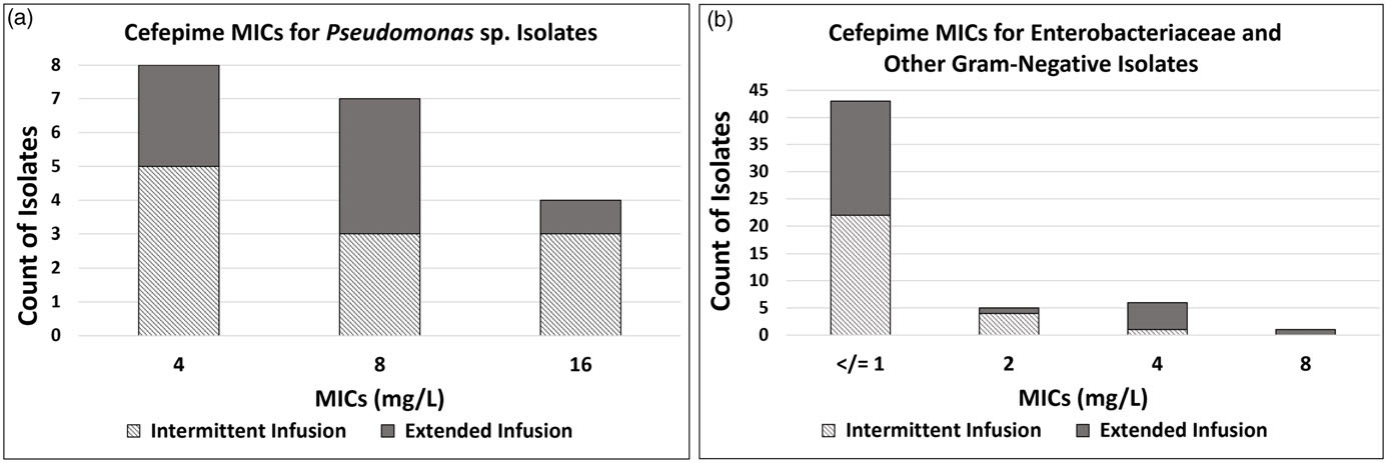
Distribution of minimum inhibitory concentrations (MICs). (A) Cefepime MICs for *Pseudomonas* spp isolates. (B) Cefepime MICs for Enterobacteriaceae and other gram-negative isolates.

In total, 204 patients met study criteria. Of the included patients, 111 (54.4%) received cefepime by intermittent infusion and 93 (45.6%) received cefepime by extended infusion. No statistical difference was noted between the baseline characteristics of the intermittent-infusion and extended-infusion groups (Table 3). Most patients had a sepsis-related MS-DRG code (intermittent infusion 73.9% and extended infusion 77.5%). The remaining patients had pneumonia-related MS-DRG codes. Severity of illness scores were major or extreme in 83.7% of patients in the intermittent infusion group and 88.1% of patients in the extended-infusion group. Rates of total positive cultures were similar in each group. MIC distributions of gram-negative organisms are shown in Figure 2. Numerically, more patients in the extended infusion group had positive blood cultures (extended infusion 47.7% vs intermittent infusion 43.1%) and positive urine cultures (extended infusion 25% vs intermittent infusion 21.6%), whereas more patients in the intermittent-infusion group had positive sputum cultures (extended infusion 22.7% vs intermittent infusion 31.4%).

**Table 3.** Demographic Characteristics of Patients Who Received Intermittent- or Extended-Infusion Cefepime

Characteristic	Intermittent Infusion (n = 111), No. (%)^a^	Extended Infusion (n = 93), No. (%)^a^
Age, median y [IQR]	65 [55.5–76.5]	67 [59–78]
Sex, male	47 (42.3)	42 (45.2)
Weight, median kg [IQR]	75.5 [60.3–93.8]	74.8 [62–92.2]
SCr on cefepime day 1 (mg/dL)	1.01 [0.74–1.55]	1.04 [0.73–1.51]
WBC on cefepime day 1 (10^3^/uL)^c^	10.8 [6.55–17.7]	12.55 [6.75–18]
Additional antibiotic therapy (fluroquinolone or aminoglycoside)^c^	11 (9.9)	5 (5.4)
Infectious diseases consultation	41 (36.9)	33 (35.5)
Cefepime duration, median d [IQR]	5 [3–7]	5 [3–7]
**MS-DRG code** ^**c**^		
177. Respiratory infections & inflammations with MCC	7 (6.3)	7 (7.5)
178. Respiratory infections & inflammations with CC	4 (3.6)	3 (3.2)
193. Simple pneumonia & pleurisy with MCC	9 (8.1)	8 (8.6)
194. Simple pneumonia & pleurisy with CC	9 (8.1)	3 (3.2)
870. Septicemia or severe sepsis with mechanical ventilation >96 h	4 (3.6)	5 (5.4)
871. Septicemia or severe sepsis without mechanical ventilation >96 h with MCC	62 (55.9)	57 (61.3)
872. Septicemia or severe sepsis without mechanical ventilation > 96 h and without MCC	16 (14.4)	10 (10.8)
**APR-DRG severity of illness score** ^**b**^,^**c**^		
4 Extreme	52 (46.8)	39 (41.9)
3 Major	41 (36.9)	43 (46.2)
2 Moderate	17 (15.3)	8 (8.6)
1 Minor	1 (0.9)	3 (3.2)
**Positive culture source** ^**c**^	51 (45.9)	44 (47.3)
Blood	22 (43.1)	21 (47.7)
Sputum	16 (31.4)	10 (22.7)
Urine	11 (21.6)	11 (25)
Wound	2 (3.9)	1 (2.3)
Other	0 (0)	1 (2.3)
**Gram-positive bacteria**	10 (9)	11 (11.8)
**Gram negative bacteria** ^**c**^	40 (36)	33 (35.4)
*P. aeruginosa*	9 (22.5)	5 (15.2)
*Klebsiella* spp	7 (17.5)	1 (3)
*Escherichia coli*	17 (42.5)	12 (36.4)
*Enterobacter* spp	2 (5)	1 (3)
*Citrobacter* spp	0 (0)	1 (3)
*Proteus* spp	2 (5)	2 (6.1)
*Burkholderia* spp	1 (2.5)	0 (0)
*Sphingomonas* spp	0 (0)	1 (3)
*Pantoea* spp	0 (0)	1 (3)
*Acinetobacter* spp	0 (0)	1 (3)
*Haemophilus influenzae*	0 (0)	1 (3)
*Stenotrophomonas* spp	0 (0)	1 (3)
*Moraxella* spp	0 (0)	1 (3)
Polymicrobial including NLF species	2 (5)	2 (6.1)
Polymicrobial not including NLF species	0 (0)	3 (9.1)

Note. IQR, interquartile range; WBC, white blood cell count; SCr, serum creatinine; MS-DRG, medical severity diagnosis-related group; MCC, major complication or comorbidity; CC, complication or comorbidity; APR-DRG, All-Patient Refined Diagnosis-Related Group; NLF, non–lactose-fermenting.

a Units unless otherwise specified.

b This report was produced using proprietary computer software created, owned, and licensed by the 3M Company. All Copyrights in and to the 3M APR DRG classification system(s) are owned by 3M. All rights reserved.

c Statistical tests did not show a significant difference between groups.

Clinical outcomes did not differ between the study groups (Table 4). Death occurred infrequently in both groups: intermittent infusion (n = 6, 2.9%) versus extended infusion (n = 3, 1.5%) (*P* = .45). Hospital and ICU length of stay did not differ, and 30-day and 90-day readmission occurred at similar rates in both groups (Table 4).

**Table 4.** Clinical Outcomes of Patients Who Received Intermittent- or Extended-Infusion Cefepime

Outcome Variable	Intermittent Infusion (n = 111), No. (%)^a^	Extended Infusion (n = 93), No. (%)^a^	*P* Value^b^
In-hospital mortality	6 (2.9)	3 (1.5)	.45
Hospital length of stay, median d [IQR]	6 [4–10]	6 [4–9]	.67
ICU LOS, median d [IQR]	0 [0–4]	0 [0–2]	1.0
**Readmission rate** ^**c**^			
30-day (any APR-DRG)	49 (44.1)	45 (48.4)	.55
30-day (same MS-DRG)	10 (9)	5 (5.4)	.32
90-day (any APR-DRG)	68 (61.3)	63 (67.7)	.34
90-day (same MS-DRG)	15 (13.5)	9 (9.7)	.40

Note. IQR, interquartile range; ICU, intensive care unit; LOS, length of stay; APR-DRG, All-Patient Refined Diagnosis-Related Group; MS-DRG, medical severity diagnosis-related group.

a Units unless otherwise specified.

b *P* < .05 is significant.

c This report was produced using proprietary computer software created, owned, and licensed by the 3M Company. All Copyrights in and to the 3M APR-DRG classification system(s) are owned by 3M. All rights reserved.

In addition to clinical outcomes, medication cost was evaluated. Utilizing an extended infusion dosing strategy (4 grams/day) led to decreased average cost per patient per day, costing $43.39, compared to the intermittent infusion strategy (6 grams/day), costing $86.06, a 50.4% reduction.

## Discussion

Multidrug-resistant organisms continue to challenge healthcare providers. New drug development is slow to progress, and the need for dosing regimens that optimize current antibiotics is growing. Extended-infusion (3–4 hours) cefepime is associated with a higher probability of target attainment at elevated MICs compared to 30-minute infusions.^10,11^ For cephalosporins, 60% ƒT>MIC has been shown to be bactericidal, with some authors recommending dosing strategies to achieve >90% ƒT>MIC.^6,10^ To our knowledge, this study is one of the first to examine clinical outcomes of extended-infusion cefepime using a dosage of 1 gram every 6 hours.

Cefepime 1 gram every 6 hours administered over 3 hours can successfully achieve adequate time above the MIC (>60%) for an MIC of 8 mg/L, the current susceptibility breakpoint for *P. aeruginosa*. This approach can be beneficial during empiric use when the pathogen is unknown. Less frequent dosing strategies may be acceptable once the MIC of the pathogen is known to be <8 mg/L and source is confirmed.

Stewardship programs are beneficial on many fronts, including improving clinical outcomes and reducing healthcare costs.^2^ Other studies have advocated for cefepime 2 grams every 8 hours administered by extended infusion despite the increased cost needed to achieve pharmacodynamic benefit.^8^ Extended infusion does require the use of a dedicated line for most of the day in addition to the need to evaluate compatibility with additional intravenous therapies. Lodise et al^14^ published on their experience implementing prolonged infusions at Albany Medical Center Hospital in 2006, noting that prolonged infusion should only be used when benefits outweigh the risks. Many institutions have switched to extended infusion as the standard of care to achieve optimal pharmacodynamics in their patients. Additionally, as in our study, extended-infusion regimens may have the benefit of utilizing less total daily medication, thus leading to potential cost savings without affecting patient outcomes. The regimen of 1 gram every 6 hours with a total dose of 4 grams per day in patients with normal renal function also has the potential benefit of reduced neurotoxicity. Although we did not evaluate neurological adverse events, other researchers have identified daily doses of >4 grams of cefepime per day as a risk factor for elevated cefepime trough concentrations and neurotoxicity.^15–17^

This retrospective chart review had several limitations. Inpatient mortality in the intermittent infusion group was numerically higher than that observed in the extended-infusion group, although this was not statistically significant. The overall low mortality rate coupled with a small sample size may have affected our ability to detect a true difference. The numerical reduction in mortality seen with extended infusion in our study is supported by findings from a similar study conducted by Bauer et al,^1^ which showed reduced mortality with extended infusion (6 grams/day) versus intermittent infusion (6 grams/day): 11 (3%) versus 1 (20%), respectively (*P* = .03). Another limitation of our study was the number of patients without a positive culture and the variability of organisms among those with a positive culture. This limited our ability to correlate a benefit of a dosing regimen against a specific organism.^1^

As a retrospective study, data collection was limited to historical documentation that occurred during the admission. After changing the standard of care to extended-infusion cefepime, UMMMC converted their electronic medical record system, which required data collection in 2 different systems. This change led to unique challenges that will likely be faced by many institutions in the coming years.

In summary, clinical outcomes, including length of stay and readmission, did not differ with cefepime dosage of 1 gram every 6 hours infused over 3 hours compared to a cefepime dosage of 2 grams every 8 hours infused over 30 minutes. However, costs may be reduced by utilizing an extended infusion dosing regimen with 1-gram vials compared to intermittent infusion with 2-gram vials. Our study adds to the growing literature supporting the concept that protocols using extended infusion of β-lactam antibiotics have similar or improved outcomes compared to protocols using intermittent infusion. Prospective multicenter high-quality studies should be conducted to evaluate the impact of cefepime extended infusion (4 grams per day) on inpatient mortality.
